# Retrospective Single Nucleotide Polymorphism Analysis of Host Resistance and Susceptibility to Ovine Johne’s Disease Using Restored FFPE DNA

**DOI:** 10.3390/ijms25147748

**Published:** 2024-07-15

**Authors:** Amanda Kravitz, Mingsi Liao, Gota Morota, Ron Tyler, Rebecca Cockrum, B. Murali Manohar, B. Samuel Masilamoni Ronald, Michael T. Collins, Nammalwar Sriranganathan

**Affiliations:** 1 Center for One Health Research, Department of Biomedical Sciences and Pathobiology, Virginia-Maryland College of Veterinary Medicine, Virginia Polytechnic Institute and State University, Blacksburg, VA 24061, USA; 2 Department of Animal and Poultry Sciences, Virginia Polytechnic Institute and State University, Blacksburg, VA 24061, USA; 3 Department of Veterinary Pathology, Tamilnadu Veterinary and Animal Sciences University, Madhavaram Milk Colony, Chennai 600051, Tamil Nadu India, India; 4 Department of Pathobiological Sciences, School of Veterinary Medicine, University of Wisconsin-Madison, Madison, WI 53706, USA

**Keywords:** paratuberculosis, FFPE tissues, DNA repair, GWAS, host susceptibility

## Abstract

Johne’s disease (JD), also known as paratuberculosis, is a chronic, untreatable gastroenteritis of ruminants caused by *Mycobacterium avium* subsp. *paratuberculosis* (MAP) infection. Evidence for host genetic resistance to disease progression exists, although it is limited due to the extended incubation period (years) and diagnostic challenges. To overcome this, previously restored formalin-fixed paraffin embedded tissue (FFPE) DNA from archived FFPE tissue cassettes was utilized for a novel retrospective case-control genome-wide association study (GWAS) on ovine JD. Samples from known MAP-infected flocks with ante- and postmortem diagnostic data were used. Cases (N = 9) had evidence of tissue infection, compared to controls (N = 25) without evidence of tissue infection despite positive antemortem diagnostics. A genome-wide efficient mixed model analysis (GEMMA) to conduct a GWAS using restored FFPE DNA SNP results from the Illumina Ovine SNP50 Bead Chip, identified 10 SNPs reaching genome-wide significance of *p* < 1 × 10^−6^ on chromosomes 1, 3, 4, 24, and 26. Pathway analysis using PANTHER and the Kyoto Encyclopedia of Genes and Genomes (KEGG) was completed on 45 genes found within 1 Mb of significant SNPs. Our work provides a framework for the novel use of archived FFPE tissues for animal genetic studies in complex diseases and further evidence for a genetic association in JD.

## 1. Introduction

Johne’s disease (JD), otherwise known as paratuberculosis caused by *Mycobacterium avium* subsp. *paratuberculosis* (MAP) infection, remains an insidious, costly, and frustrating issue for scientists, veterinarians, and livestock producers alike. This disease results in economic losses between 250 million and 1.5 billion dollars annually in the U.S. [1]. This chronic disease is significant in both cattle and small ruminant (sheep and goat) production systems due to decreased milk production and premature culling of affected animals. Animals are primarily infected as neonates through fecal-oral contamination from the environment or contaminated milk from a dam. Initial infection is followed by an extended incubation period of 1–8 years before progressing to clinical disease characterized by progressive weight loss and hypoproteinemia [2]. However, approximately 90% of infected hosts do not progress to clinical disease and instead remain asymptomatic for life, while only 10% progress to clinical disease [2,3,4]. The majority of animals that remain asymptomatic are capable of intermittent shedding of MAP in feces, contaminating the environment and thus the infection cycle. Further, compared to cattle, clinically affected sheep and goats do not exhibit classical chronic diarrhea, which helps in diagnosis and control decisions [5]. This species-specific difference in clinical disease presentation makes identifying and removing infected sheep and goats more challenging for producers and warrants further investigation into mechanisms of control and minimizing the prevalence of JD in these species.

In the past two decades, there has been an increased focus on investigating host genetic polymorphisms associated with differences in MAP exposure. This effort is aimed at understanding molecular mechanisms associated with natural protection from infection as well as identifying markers to use for selective breeding. Selective breeding for resistance or decreased susceptibility to MAP infection has the potential to aid in current test and cull control strategies, resulting in a decreased prevalence of MAP infection over time. This has led to studies utilizing multiple methods of genetic analysis, including candidate gene studies, genome-wide association studies (GWAS), genome-wide expression analysis (GSEA), and the identification of SNPs, candidate genes, and pathways involved in resistance/susceptibility [6,7]. The bulk of the available studies have been conducted on dairy cattle, primarily in Holsteins and Jersey breeds, representing the most common dairy breeds [8,9,10,11,12,13,14,15]. Fewer studies have been conducted thus far on sheep using these genetic association methods to identify markers of resistance/susceptibility to JD [16,17,18,19].

The results identified in genetic association studies on JD in ruminants tend to result in new variants and genes, with few validating previously identified SNPs and candidate genes. These differences could be due to the differences in populations, genomic models, diagnostic tests, and JD terminology used to classify cases vs. controls [4]. Despite this, there are candidate genes and functional pathways that overlap between studies in JD, as well as those for *Mycobacterium tuberculosis* (Mtb) infection and Crohn’s disease (CD) in humans. Some of the most studied genes and encoded proteins in JD resistance/susceptibility include; Solute carrier family 11 member 1 gene (*SLC11A1)*, Nucleotide-binding-oligomerization domain containing gene 2 (*NOD2)*, Toll-like receptor genes (*TLR 1*, *2, 4*, *9*), and Major histocompatibility complex type II genes (*MHC-II*), which are found across breeds and species [6]. Despite the lack of consistent identification of common SNPs and candidate genes between studies, significant overlap in the functional pathways identified exists across studies and species. These pathways include the NOD-like receptor signaling pathway, the Toll-like receptor signaling pathway, the NF-kappa B signaling pathway, the MAPK signaling pathway, the TNF signaling pathway, and the Wnt signaling pathway, to name a few [20,21,22,23]. These pathways and genes within these pathways primarily function in the innate immune recognition and initiation of downstream inflammatory signaling in response to intracellular bacteria, specifically mycobacteria. Identification of common genes between CD, JD, and Mtb infection indicates possible common functional pathways associated with host resistance/susceptibility to disease [24,25,26,27]. 

The majority of studies investigating host genetic influence in MAP infection are done using a single breed, and to the best of our knowledge, they are no studies using archived formalin-fixed paraffin-embedded (FFPE) tissue-derived DNA. Here, we build upon previous work validating successful restoration of FFPE-derived DNA and genotyping utilizing Illumina OvineSNP50 BeadChip [28] to conduct a case-control GWAS to identify SNPs, genes, and functional pathways associated with resistance/susceptibility to MAP infection in sheep. We provide evidence that GWAS studies utilizing restored FFPE-derived DNA were able to identify significant SNPs in this case-control study and show the untapped potential of FFPE tissues in complex disease studies. The SNPs, genes, and pathways identified here add to the current knowledge of host immunity to MAP infection in sheep. This work further validates and supports the future utility and clinical relevance of FFPE tissues in livestock genetic studies and veterinary medicine as a whole. 

## 2. Results

### 2.1. MAP Diagnostics

Upon reviewing the MAP diagnostic results conducted previously at TANUVAS for the Kattupakkam Red sheep, only four animals were found to be positive upon tissue qPCR. One sheep was tissue qPCR negative (JD656) but had 2+ acid-fast Bacilli (AFB) found in small intestine (SI) samples. The remaining 25 Kattupakkam Red sheep had negative tissue qPCR results and no AFB in the tissues examined. The four samples tested by Wisconsin Veterinary Diagnostic Laboratory (WVDL) were all found to be tissue qPCR positive; two sheep (15111 and 4106) had AFB identified in SI samples (graded) as 2 and 3, respectively. Full diagnostic results, age, and breed of each animal can be found in Supplementary Table S1.

### 2.2. Case-Control Definitions

In order to maintain consistent case definition criteria in JD genetic studies, the terminology previously proposed was used to formulate our case and control definitions [4]. Although multiple diagnostic tests, including serum antibody testing and culture, were obtained for some of the included samples, these tests could not be used in our definitions due to the variation in available results in this retrospective study. Table 1 shows case and control definition criteria and sample breakdown.

Using the diagnostic information available and case definitions, sheep were categorized as a case or control using tissue qPCR and tissue AFB results according to the proposed definitions. Cases were defined as sheep positive on tissue qPCR or negative on qPCR but with AFB in tissues. Controls were sheep that tested negative upon tissue qPCR and had no AFB in tissues. These definitions resulted in nine cases and 25 controls to be included in the case-control analysis.

### 2.3. Genotyping QC

Sample and variant pruning were conducted in both Genome Studio and Plinkv1.9. SNPs with call rates <0.9 were removed, leaving 29577 SNPs passing QC and FFPE samples with call rates <0.8 being removed, and SNPs with MAF < 0.01 were also removed. This left FFPE sheep passing QC with 6 males, 28 females, and 29,577 SNPs for analysis, where N = 9 were cases and N = 25 were classified as controls passing QC.

### 2.4. GEMMA Case-Control Analysis

**Figure 1:**
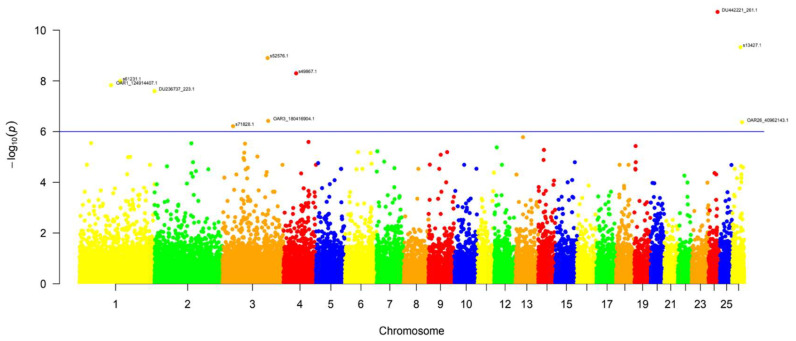
**Manhattan plot *p* < 1 × 10^−6^** showing the distribution of the 10 significant SNPs identified above the calculated genome-wide significance threshold of *p* < 1 × 10^−6^. Figure generated using the RStudio Package qqma. Each chromosome is located on the *x*-axis, and statistical significance on *y*-axis, each individual SNP is represented as a single dot along a given chromosome.

**Figure 2:**
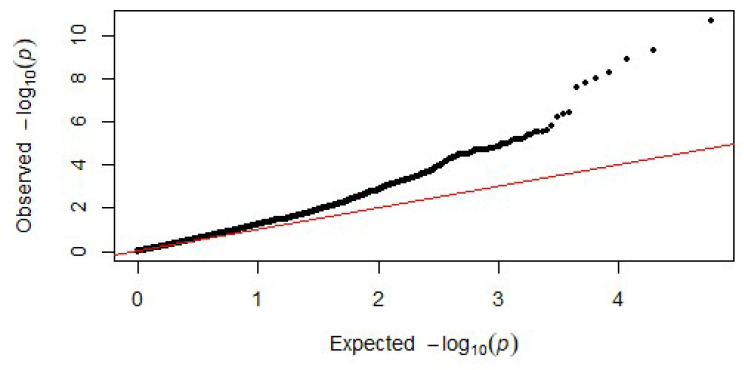
**Q-Q Plot**: Observed vs. expected *p*-values are plotted and were made in the RStudio Package: qqma. Showing expected vs. observed is a graphical representation of the deviation of the observed *p*-values from the null hypothesis indicating few SNPs are in linkage-disequilibrium with a causal polymorphism resulting in small *p*-values.

Using GEMMA and a case-control analysis between infected cases (N = 9) and non-infected controls (N = 25) resulted in 10 SNPs above the calculated significance level of *p* < 1 × 10^−6^, which was above the Li and Ji calculated threshold of *p* < 4.001 × 10^−5^. The threshold used is closer to that of the Bonferroni correction threshold (1.69 × 10^−6^). The results can be visualized using the Manhattan plot in Figure 1. A graphical representation of the deviation of the observed *p*-values from the null hypothesis can be found in Figure 2. The 10 SNPs belong to the following chromosomes; chromosome 1 (3 SNPs), chromosome 3 (3 SNPs), chromosome 4 (1 SNP), chromosome 24 (1 SNP), and chromosome 26 (2 SNPs). The calculated *p*-values range from 6.12 × 10^−7^ to 1.93 × 10^−9^; see Table 2 for detailed results per SNP*. The most significant identified SNP was found on chromosome 24:33324074, with a *p*-value of 1.92 × 10^−11^ and was the only SNP identified at this significance level in chromosome 24. The second most significant SNP was found on chromosome 26:30765074, with a *p*-value of 4.70 × 10^−10^. Of the SNPs found on chromosome 1, all had *p*-values from 1.01 × 10^−8^ to 2.52 × 10^−8^. The SNPs closest together were found on chromosome 3, however, they were still over 1 Mb apart.

#### Gene Identification from Significant SNPs

For each of the 10 identified SNPs, a window of 1 Mb on the NCBI genome data browser *Oarv4.0* genome assembly was used to find surrounding genes. A full table of identified genes and functions from NCBI can be found in Supplementary Table S2, as well as a compressed table with the gene symbols for each SNP listed in Table 3. Of the 10 significant SNPs, 6 were located within a gene. Two of the three SNPs on chromosome 1 were located within genes rs401362015 (*RAB5A*—RAB5A member RAS oncogene family) and rs407060336 (*TMCO1*—transmembrane and coiled domains 1). All three SNPs found on chromosome 3 were located within a gene: rs428083866 (*ANTXR1*-ANTXR cell adhesion molecule 1), rs401844951 (*ANKS1B*-Ankyrin repeat and sterile alpha domain containing 1B), and rs399773060 (*CFAP54*-Cilia and flagella associated protein 54). One of the SNPs on chromosome 26 was also found within the gene rs415353783 (*KCNU1*-Potassium calcium-activated channel subfamily U member 1). Of the SNPs not located within a gene, one SNP rs410166885 on OAR 1 had no known genes within the 1 Mb window, and the remaining three SNPs had 6 genes (rs406625389 OAR 4), 18 genes (rs55627888 OAR 24), and 15 genes (rs399723913 OAR 26). The most significant SNP on OAR 24 had the most genes (18) within the 1 Mb window, and the second most significant SNP found on OAR 26 was located within the *KCNU1* gene. 

In total, 45 genes were identified within a 1 Mb window from the 10 significant SNPs found in the case-control analysis (Table 3). These genes and encoded proteins were then used to determine the functional relationship to MAP infection as well as the associated pathways these genes belong to.

### 2.5. Gene Ontology and Pathway Analysis

The identified 45 gene symbols were placed into PANTHER, where gene ontology (GO) groups were identified based on human ortholog data for biological processes. There was no statistical significance for GO enrichment; however, information was used to further interrogate the function of the identified genes. In total, 11 different GO biological processes contained at least one of the identified genes (43 total genes identified in PANTHER and 80 total process hits after filtering unclassified). Cellular processes (GO: 0009987) contained the most genes (26), followed by metabolic processes (GO: 0008152), contained (12), biological regulation (GO: 0065007), and localization (GO: 0051179), both with 11 genes each. No biological processes were found to be directly related to the immune system.

These same 45 genes were then put into PANTHER pathways to identify functional pathways based upon human orthologs and conserved functional pathways. Again, no statistical significance was found between any of the 19 different pathways identified from 43 genes and 22 total pathway hits. All but two pathways contained only one gene. Nicotine pharmacodynamics pathway (P06587) contained two genes (*CHRNA6, CHRNB3*), and Nicotinic acetylcholine receptor signaling pathway (P00044) contained three genes (*STX1A*, *CHRNA6*, *CHRNB3*). Of the identified pathways, eight were directly related to immune system function: the apoptosis signaling pathway (P00006), the interleukin signaling pathway (P00036), inflammation mediated by the chemokine and cytokine signaling pathway (P00031), the PDGF signaling pathway (P00047), B cell activation (P00010), the Wnt signaling pathway (P00057), the Toll receptor signaling pathway (P00054), and T cell activation (P00053). Only the gene *IKBKB* was found in seven of the eight related immune pathways, and *FZD9* was the only gene found in the Wnt signaling pathway. 

Due to the lack of statistical significance and the number of genes without hits from PANTHER, KEGG pathway analysis was also utilized. To prune the number of genes to input into KEGG to determine ovine-specific pathways, genes found in relevant PANTHER pathways (*IKBKB*, *STX1A*, *CHRNA6*, *CHRNB3*, *FZD9*, *PLAT*, *CLDN4*, *CLDN3*, *VPS37D*, *VDAC3*) were included in addition to the 6 genes found to harbor SNPs within (*RAB5A*, *TMCO1*, *ANTXR1*, *ANKS1B*, *KCNU1*, and *CFAP54*). A total of 16 genes were then imputed into KEGG to determine ovine specific pathways. 

From the 16 genes, a total of 74 different ovine-specific pathways were found a full list of the 74 pathways and associated genes can be found in Supplementary Table S3. The most genes per pathway found here were four genes belonging to pathways of neurodegenerative-multiple diseases (oas05022): *RAB5A*, *STX1A*, *FZD9*, and *VDAC30*. There were three pathways with three genes each, including the NOD-like receptor signaling pathway (oas04621), Alzheimer disease (oas05010), and Hepatitis C (oas05160). The NOD-like receptor signaling pathway contains the genes *ANTXR1*, *IKBKB*, and *VDAC3* and is directly involved in the host response to mycobacteria. Additionally, this pathway and the *NOD2* gene are well-known in terms of host resistance/susceptibility to MAP infection, CD, and Mtb infection. The pathways were further investigated by the number of genes within the pathway and their physiological relatedness to MAP infection. We found seven pathways with two genes and a direct relationship to MAP infection. A table complete with MAP-associated pathways can be found in Supplementary Table S4. These pathways include Ras signaling pathways (oas04014), endocytosis (oas04144), Salmonella infection (oas05132), mTOR signaling (oas04150), cell adhesion molecules (oas04514), tight junction (oas04530), and leukocyte trans endothelial migration (oas04670). We also identified 17 pathways with only one gene but with a function in the MAP immunity/immune system, including six related to immune signaling pathways: RIG-I-like receptor signaling, MAPK signaling pathway, chemokine signaling pathway, IL-17 signaling pathway, TNF signaling pathway, and NF-kappa B signaling pathway. These 17 pathways also included the phagosome (oas04145) and tuberculosis (oas05152), both of which contain the *RAB5A* gene and are directly related to MAP infection. Further, Toll-like receptor signaling (oas04620) and the Wnt signaling pathway (oas04310) were both found in this group and were also identified in the PANTHER pathway analysis and known to function in MAP immunity. We also found pathways in the adaptive immune system, including Th1 and Th2 cell differentiation (oas04657), Th17 differentiation (oas04659), the T-cell receptor signaling pathway (oas04660), and B-cell receptor signaling (oas04662). From the KEGG analysis, we also found eight pathways with two genes but no relationship to MAP infection or directly related to the immune system. 

Utilizing the results obtained in both the PANTHER and KEGG analyses, further pruning of genes (Table 4) and pathways (Table 5 and Table 6) was conducted to find those with the most functional relatedness with host response to mycobacteria and MAP, specifically using available literature. Pruning resulted in four potential functional candidate genes belonging to the eight pathways involved in innate immunity and inflammatory signaling, and four associated with the adaptive immune system. 

## 3. Discussion

The purpose of this study was to utilize genotype data obtained from restored FFPE-derived ovine DNA to use as a case-control GWAS for resistance/susceptibility to ovine JD. Samples used were from sheep with ante- and post-mortem MAP diagnostic data, which increased confidence in classifying included animals as truly exposed to MAP. To further increase our case-control analysis, the definitions used here followed those proposed to increase consistency across genetic studies of JD, as this was identified as a major inhibiting factor in the lack of cross study validation [4]. Cases in this study were defined as known exposed sheep between the ages of 2–7 years old with evidence of MAP in the tissues (qPCR or culture positive or negative but with AFB found), indicating these cases as ‘infected’ compared to controls. Controls in comparison were animals with known exposure; there was no evidence of MAP in tissues (qPCR and tissue culture negative, without AFB in tissues), indicating controls as ‘not-infected’ compared to cases. The terminology utilized here provides increased confidence in the diagnostic classification of animals in this study, thus increasing the strength of the biological relevance. Due to the ease of sampling and testing using serum antibody ELISA, many studies have used this single test to classify cases and controls. In JD, animals progress to clinical disease despite the presence of MAP-specific antibodies, indicating antibody presence is not protective and is also shown to not correlate with stage of infection [4,29]. Although results from studies utilizing ELISA as the sole diagnostic criteria can shed light on differences in the ability to mount an antibody response, ELISA is not shown to be sensitive or specific enough to be used alone in the diagnosis of JD, especially in sheep [5]. Despite this effort to ensure accurate diagnostic terminology, our study is limited by the small number of cases (9) and controls (25) used here. Although efforts to increase sample size were made, we were unsuccessful in obtaining ovine FFPE samples that matched our robust case-control criteria. This limitation is innate to retrospective studies and those investigating complex or polygenic diseases for which unequal distributions of cases and controls occur, as is the case with JD. Similar GWAS studies on livestock species for disease resistance have utilized similar-sized groups [30,31]. 

The results presented here identified 10 SNPs reaching genome-wide significance, associated with 45 total genes. The top three most significant SNPs were found in OAR 24 (rs55627888), OAR 26 (rs415353783), and OAR 4 (rs406625389). Although none of the SNPs have been previously found in other genetic association studies on JD, the chromosomes we identified as significant SNPs have also been found to be significant. Specifically, in this study, 50 positive and 50 negative Italian sheep were identified using serum ELISA, and significant SNPs were found on OAR 24, 26, 4, 3, and 1, which we also identified [17]. However, the specific location on each chromosome was different and challenging to interpret given the two different genome assembly versions used between studies. Another study in sheep identified *TLR2* haplotypes encoding Q650 with reduced susceptibility to JD in Turkish sheep [18]. However, we did not identify any SNPs on OAR 17, where the TLR2 gene is located. However, TLR-2 also functions within the identified significant pathways related to innate immune recognition and response to intracellular bacteria. Of the total 45 genes identified here, we found four potential candidate genes: *IKBKB*, *FZD9*, *ANTXR1*, and *RAB5A*. Of these identified genes, *IKBKB* was previously identified in a GSEA-SNP study as a leading edge gene [32]. The remaining genes identified here are shown to have functional roles within the pathways found, although they have not been identified as significant in resistance/susceptibility in JD to the best of our knowledge thus far. 

The single gene *IKBKB* was found to be a leading edge gene within the RIG-I/MDA5-mediated induction of IFN alpha beta pathways in the previous GSEA-SNP study on Holstein cattle mentioned previously [32]. This gene plays a central role in the activation of the NF-kappa B signaling pathway, aiding in the initiation of pro-inflammatory signaling in response to MAP infection, including TNF-alpha. Not only was *IKBKB* found significant in our study, but it was also the single gene with the most hits from the KEGG pathway analysis belonging to 56 total pathways. *IKBKB* was also found as the sole gene found within both the innate and adaptive immune pathways in the KEGG analysis, indicating this gene may play a vital role in both the innate and adaptive response to MAP infection by acting as a bridge in functions. IKBKB is also involved in Toll-like signaling pathways, where the sensing of pathogen associated molecular patterns (PAMPs) like those from MAP through TLR-2 results in downstream NF-kappa B activation through IKBKB [33,34]. Our results support the importance of this gene and associated pathways in response to MAP and its role as a potential candidate gene for MAP resistance/susceptibility. IKBKB and NF-kB signaling further recruit T-cells to the site of infection, where antigen presentation to CD4+ T-cells can lead to IFN-gamma expression, which has been identified as vital in the host response to intracellular bacteria [35,36]. Recently, IFN-gamma responses were used as a measure of robust response to PPD in a study investigating gene expression changes [37]. Thus, IKBKB function is vital to the host response to MAP, in both the innate and adaptive branches of the immune system.

Of the other candidate genes, *RAB5A* was identified as significant, containing the identified SNP rs428083866 on OAR 3. Although not identified previously in MAP resistance/susceptibility studies, the *RAB5A* gene product was found to be involved in the phagosome and tuberculosis KEGG pathways. This gene product functions in the early endosome, is thought to be involved in phagosome maturation, and regulates early endosome fusion [38]. Blocking the function of RAB5A has been shown to result in the arrest of phagosome maturation in mycobacterial infections [39]. This family of Rab proteins is also identified as functioning in autophagy in the context of Mtb infection and was identified as a potential target gene for immunotherapy [40].

The *ANTXR1* gene was also identified in our analysis and was found to be involved in the NOD-like receptor signaling pathway, one of the most identified pathways in relation to MAP immunity. This gene is also involved in the Wnt/Beta Catenin signaling pathway and has been identified in gastric cancer, thus providing a role for this gene in intestinal inflammatory signaling and regulation. It was recently identified as a prognostic biomarker correlating with stromal and immune cell infiltration in gastric cancer [41]. This study identified that ANTXR1 has immunosuppressive functions, promotes the secretion of immunomodulatory factors, and is found in the M2 macrophage polarization pathway and T-cell exhaustion [41]. Similarly, *CLDN4* and *CLDN3* both belong to the claudin family of proteins and act as vital members in maintaining tight junction function, where dysfunction of these genes (specifically *CLDN4*) has been implicated in prolonged intestinal inflammation as well as in human CD [42,43]. The role of both chronic intestinal inflammation and function of intestinal tight junctions and cell adhesion are also seen in JD, where MAP invasion across the intestinal epithelium is observed, and CLDN4 has been identified as differentially expressed in a study utilizing transcriptional profiling of tissue samples from MAP infected vs. subclinical Holsteins [44]. 

Although not directly identified in a GWAS in relation to host susceptibility/resistance to JD, *KCNU1* was recently reported to be found to be differentially methylated in an in-vitro study in Chinese Holstein cattle [20]. This study collected PBMCs from infected and non-infected cattle and exposed the macrophages to MAP, investigating the differential host gene expression and methylation at various time points. The *KCNU1* gene was found to be within the top 10 differentially methylated genes in clinical (infected) cattle compared to subclinical cattle (fecal qPCR negative but ELISA positive). Additionally, this work also found the FZD7 gene, a member of the same frizzled class receptors as FZD9 identified here, was shown to be in the top 10 differentially methylated genes when subclinical cattle were compared to healthy controls (negative fecal qPCR and negative serum ELISA) [20]. This class of proteins serves as receptors for the Wnt family of proteins, which together are involved in numerous vital processes, including immune regulation [45]. Interestingly, this pathway and specific members of the WNT family have been identified previously as proposed candidate genes for resistance/susceptibility to JD and Mtb infection and chronic inflammation [23,46,47]. Further, the *WNT2* gene was identified as a candidate gene for resistance to MAP in Holstein cattle using both fecal culture and antibody response to MAP in 162 cases and 162 controls, and the specific polymorphism identified, located in the promoter region, is associated with paratuberculosis susceptibility [23]. Together, this indicates a role for members of the Wnt signaling pathway in the host response to MAP, where FZD9 should be further investigated as a possible candidate gene for resistance/susceptibility to MAP infection.

The identified pathways found to be most directly involved in host response to MAP are the nine pathways found to have a relationship to innate immune recognition of MAP and downstream inflammatory signaling (NOD-like receptor signaling, cell adhesion, phagosome, tuberculosis, NF-kappa-B signaling, Toll-like receptor signaling, Wnt signaling, IL-17 signaling pathway, and TNF signaling pathway). This supports previous studies indicating the vital role of innate immune fitness in response to MAP, where it is hypothesized that host-pathogen interactions occurring in early infection set the stage for the entirety of infection [3]. Further, if a host harbors SNPs influencing the expression or function of genes and innate immune pathways, this can lead to a delay in pro-inflammatory signaling and MAP antigen presentation, resulting in a delay in MAP-specific activation of the adaptive immune system. This delay, along with the MAP-mediated manipulation of host macrophages, can give MAP an upper hand where the host immune system is unable to catch up to contain infection, resulting in clinical disease [2,6]. Here we find *IKBKB* to be linked to the adaptive immune system through four pathways (Th1-Th2 differentiation, Th17 differentiation, T-cell receptor signaling, and B-cell receptor signaling), further supporting the role of *IKBKB* in branching innate and adaptive host responses to MAP and should be investigated in future studies.

Together, the pathways identified here overlap with those identified previously in other studies of JD genetics, including the NOD-like receptor signaling pathway, phagosome, tuberculosis, NF-kappa B signaling, Toll-like receptor signaling, TNF signaling, Wnt signaling, and IL-17 signaling [7,48,49]. The candidate genes identified here, in addition to *NOD2*, *SLC11A1*, and *TLRs*, function within these pathways to initiate a robust immune response to MAP. These conserved pathways function together and contain overlapping genes identified here and identified in previous genetic association studies in JD, as well as CD and Mtb infection, which could indicate a possible common pathophysiology between these diseases [50,51,52]. Further, upon ingestion of food or water contaminated with MAP, it is translocated from the intestinal lumen via interactions with microfold-cells (M-cells) and intestinal epithelial cells [53]. Recognition of MAP pathogen-associated molecular patterns (PAMPS) can occur via intracellular NOD2 or transmembrane TLRs binding N-glycolyl MDP, mycobacterial peptidoglycan, or mycobacterial lipoproteins (TLR2) specifically [48,54]. This results in downstream activation of NF-kappa B and MAPK, including TNF-alpha, IL-6, and IL-18, producing an anti-inflammatory response to MAP [55]. SLC11A1 is actively recruited to the phagosome membrane, leads to increased MHC-II and TNF-alpha expression, and has been shown to inhibit intracellular growth [56]. Production of a rapid and robust pro-inflammatory response leads to rapid recruitment of T-cells capable of interacting with infected macrophages through antigen presentation, resulting in the induction of a cell-mediated immune response to MAP [2]. 

This study, to the best of our knowledge, is the first to utilize ovine FFPE-derived DNA for a disease GWAS, setting the stage for future work to build upon the methods and results described here. Additionally, the limitations, including the small sample sizes, distribution of breeds, and unequal number of cases and controls, hinder the ability to directly extrapolate the results presented here. However, despite these limitations, this work is invaluable and groundbreaking in both the DNA type used and the retrospective case-control analysis for GWAS in ovine JD, for which studies are lacking. These sample types are not only vital to histopathology and post-mortem diagnostics; FFPE tissue blocks are stable at room temperature for years, maintaining the tissue architecture as well as the genetic material within. Further, the ability to freely distribute these sample types internationally without inhibitory regulations makes FFPE tissues a great sample for international collaborative retrospective studies. However, the use of these sample types and the retrospective case-control study design have innate limitations we were unable to overcome, including small sample sizes, unequal numbers of cases and controls, and unequal distribution of breeds between cases and controls. Despite our best efforts to identify samples of the same breed and meet case-definition criteria, we were unable to increase our sample size or distribute breeds evenly between groups. This does limit the statistical significance of this work, and interpretations from the results should be used with caution until future validation using a larger sample set can be conducted. The *p*-value threshold used here (*p* < 1 × 10^−6^) is within the levels found in other GWAS studies of ruminants to investigate JD susceptibility, where a variety of genetic models are used but significance levels of between (*p* < 1 × 10^−4^ to *p* < 1 × 10^−8^) of which our results lie within, although methods between studies vary [17,57,58,59]. 

## 4. Materials and Methods

### 4.1. Animals and Sample Collection

This project took place at Tamil Nadu Veterinary and Animal Sciences University (TANUVAS) in Chennai, India, and aimed to screen flocks of Kattupakkam Red sheep to identify ‘infected’ animals to test an anti-mycobacterial drug compound called Myoconda. The screening process took place prior, and MAP diagnostic testing, including fecal culture and PCR, continued through the 6 weeks of the study. Despite all included animals being fecal qPCR or culture positive prior to and during the study, upon necropsy and tissue processing, only four had evidence of tissue infection, either by tissue qPCR positive results or acid-fast bacilli in tissues. All animals remained asymptomatic for the entirety of the project despite having continued fecal culture and/or PCR positive results, and no differences in levels of MAP were found between any of the groups. This lack of tissue-level evidence of MAP infection in most of these sheep, despite continued positive MAP diagnostic tests, led us to pursue the genetic variation between these hosts, where exposure to MAP led to different infection outcomes in these 35 Kattupakkam Red sheep. Samples available from this project were limited to formalin-fixed paraffin-embedded (FFPE) tissue cassettes originally processed in 2007. To increase sample sizes, collaboration with other universities was needed, which led to a total of 48 FFPE ovine samples with varied MAP diagnostic criteria. After screening for samples meeting the Kattupakkam Red sheep case-control criteria and diagnostic testing, a total of 34 samples, consisting of 25 controls and nine cases, were used in this study. 

FFPE samples were obtained, restored, and validated on the Illumina OvineSNP50 BeadChip as described previously [28]. Briefly, a total of 48 ovine FFPE samples from 5 flocks were obtained, where breed, age of animal, and FFPE sample varied depending on available information (see Supplementary Table S1 for full details). FFPE samples meeting the diagnostic criteria were included in the association analysis; this includes all 35 FFPE samples from Kattupakkam Red sheep (also known as Madras Red) from Tamil Nadu Veterinary and Animal Sciences University (TANUVAS) in Chennai, India. These animals belong to a larger flock native to the region with a historical endemic issue with MAP infection. FFPE cassettes from this study were brought back to the U.S. from India. To increase the sample size, 5 additional ovine FFPE samples were obtained that met the case definition criteria: 1 was gifted by Dr. Ramachandran from Oklahoma State University, and 4 were shared via a collaboration with Dr. Michael Collins of the University of Wisconsin-Madison. 

The Kattupakkam Red sheep tissue samples used for MAP diagnostic testing included mesenteric lymph nodes and intestinal tissue samples, which were collected using aseptic techniques and divided into two equal representative portions for culture and histopathological studies. One part of the sample was refrigerated in a sterile leak proof container (for culture) and the other in 10% buffered formalin (for histopathology). The four samples from Dr. Collins were processed and tested at the Wisconsin Veterinary Diagnostic Laboratory, which houses the Johne’s Testing Center. This included tissue processing for histopathology, where mesenteric lymph nodes and small intestine samples were fixed in 10% buffered formalin before histopathology. All included animals were 2 years of age or older; for full details, see Supplementary Table S1. 

### 4.2. MAP Tissue Culture

The Kattupakkam Red sheep tissue samples were processed and cultured at TANUVAS in Chennai, India, where two grams of tissue trimmed of fat were finely chopped using a sterile scalpel blade or scissors and homogenized in a stomacher for 1 min in 25 mL of 0.9% HPC. The samples were allowed to stand at room temperature for 30 min so that foam dissipated and larger pieces of tissue settled. The tissue homogenates were poured into centrifuge tubes, taking care to avoid carrying over fat or large tissue pieces, and allowed to settle for 30 min. Ten milliliters of cellular suspension were transferred into a 30 mL centrifuge tube and incubated for 3 h at 37 °C. Then the suspension was centrifuged at 900× *g* for 30 min, and the supernatants were discarded. The pellet was resuspended in 1 mL of VAN solution and incubated overnight at 37 °C. The decontaminated fecal and tissue sediments (approximately 1.6 mL) were divided into four parts, and approximately 400 μL (8 drops) was inoculated into three tubes of Herrold’s egg yolk medium (HEYM)/Middlebrook 7H9 with mycobactin J separately and one tube of medium without mycobactin J. The culture tubes were incubated at 37 °C for 3–9 months and examined for growth fortnightly.

The four samples from Dr. Collins were processed and tested at the Wisconsin Veterinary Diagnostic Laboratory, which houses the Johne’s Testing Center, using the Johne’s liquid culture system (VersaTREK, TREK Diagnostics, Independence, OH, USA) following an AAVLD validated method. In brief, 2 g of feces were mixed with 35 mL of sterile water, vortexed, and incubated at room temperature (RT) for 30 min. A 5 mL aliquot of supernatant were removed from the middle and transferred to 2 mL of bovine brain heart infusion (BHI) broth with 0.6% Hexadecylpyridinium Chloride Monohydrate (HEX) and were incubated at 36 ± 2 °C for 18–24 h. Samples were centrifuged at 3000× *g* for 20 min, the supernatant was discarded and the pellet was resuspended in 1 mL of BHI with antibiotic brew (1 mg/mL nalidixic acid, 5 mg/mL amphotericin B, 100 mg/mL vancomycin) and incubated at 36 ± 2 °C for 18–24 h. For tissue samples, 2 g of tissue were stomached (Lab-Blender Stomacher 80, Tekmar Co., Cincinatti, OH, USA) with 5 mL of sterile water. A 20 mL volume of BHI-HEX was added and allowed to incubate at RT for 30 min. 10 mL of supernatant was removed and incubated at 36 ± 2 °C for 3 h, centrifuged at 900× *g* for 30 min, and the supernatant was removed. The pellet was resuspended in 1 mL BHI with antibiotic brew and incubated at 36 ± 2 °C for 18–24 h. Both fecal and tissue samples in BHI with antibiotic brew were cultured using the manufacturer’s protocol (VersaTREK) for 56 days, along with a positive control sample prepared in the same manner. Upon completion of incubation, slides were prepared and acid-fast stained for semi-quantification of acid-fast rods. Additionally, 500 uL of each liquid culture sample were used for RT-PCR confirmation, as described above. Positive cultures were also incubated on Herrold’s Egg Yolk Agar with Mycobactin J and an ANV antibiotic mixture (BD, Franklin Lakes, NJ, USA) at 36 ± 2 °C 16 for weeks.

### 4.3. MAP DNA Extraction

Tissue samples (MLN and intestine) from TANUVAS were processed according to the QIAGEN Tissue DNA extraction protocol using 10–25 mg of starting tissue. The extraction of *Mycobacterium avium* subsp. *paratuberculosis* DNA from liquid HEYM was carried out as described by Whittington et al. (1998), with modifications [60]. The egg yolk in HEYM culture was precipitated by adding 200 µL of culture to 500 µL absolute ethanol and allowed to stand for 2 min. They were centrifuged at low speed (8× *g* for 10 min) to deposit egg yolk on the wall of the tube. The supernatant was again centrifuged at high speed (18,000× *g* for 5 min), and the resulting pellet was washed twice in PBS and subjected to DNA extraction using the DNeasy tissue kit. The bacterial pellet was resuspended in 180 µL of lysis buffer (ATL). To this, 20 µL of proteinase K (QIAGEN INC., Hilden, Germany) was added, mixed by vortexing, and incubated at 55 °C for 1–3 h until the culture was completely lysed. To this binding buffer, AL (200 µL) was added, mixed by vortexing, and incubated at 70 °C for 10 min. Ethanol 200 µL (96–100%) was added to the sample and mixed thoroughly. The mixture was added to the DNeasy mini spin column placed in a 2 mL collection tube and centrifuged at 6000× *g* (8000 rpm) for 1 min. The flow-through was discarded. The DNeasy mini spin column was placed in a new 2 mL collection, and 500 µL of wash buffer 1 (AW1) was added and centrifuged for 1 min at 6000× *g* (8000 rpm). The flow-through was discarded. The DNeasy mini spin column was placed in a new 2 mL collection, and 500 µL of wash buffer 2 (AW2) was added and centrifuged for 3 min at 20,000× *g* (14,000 rpm). The flow-through was discarded. The DNeasy mini spin column was placed in a clean 1.5 mL microcentrifuge tube and 200 µL of elution buffer (AE) was pipetted onto the DNeasy membrane. The column was incubated for 1 min at room temperature and centrifuged at 6000× *g* (8000 rpm) for elution. 

Tissue and liquid culture samples were extracted with an internal control using a magnetic particle bead-based processor (Kingfisher, Thermo Fisher Scientific, Vanta, Finland) and extraction kit (MagMAX Total Nucleic Acid Isolation Kit, Thermo Fisher Scientific). All protocols were conducted according to kit instructions and have been validated at WVDL in accordance with AAVLD guidelines. Eluted DNA from samples regardless of origin were held at −20 °C until use on subsequent MAP PCR. 

### 4.4. FFPE DNA Extraction

Full FFPE DNA extraction materials and methods can be seen in more detail as described previously [28]. Briefly, extractions were done according to the Qiagen FFPE DNA Extraction kit protocol (QIAGEN INC., Germeny) using 25 mg of FFPE tissue per sample. Tissue sections 5–10 μM thick per sample were used, and xylene was used to remove paraffin wax before continuing the Qiagen DNA extraction protocol. DNA quality and quantity were checked via the Thermo Fisher NanoDrop 1 Spectrophotometer (Thermo Fisher Scientific, Waltham, MA, USA). DNA concentrations and 260/280 and 260/230 ratios were recorded, and samples were then held at −20 °C until use.

### 4.5. MAP DNA Detection

The 35 Kattupakkam Red sheep tissue samples (and liquid culture samples) were subjected to qPCR to identify the target, which is the single-copy MAP gene *hspX,* run on the BioRad CFX Connect Real-Time PCR Thermal cycler (Bio-Rad, Hercules, CA, USA). Samples were run using positive and negative tissue-matched controls and compared to a validated 5 series MAP standard curve. For this assay, test samples of 6 μL DNA were added to 54 μL of the provided qPCR master mix with an incorporated fluorogenic probe and loaded into duplicate wells at 25 μL reaction volume into a 96-well plate. An initial enzyme activation step for 10 min at 95 °C is followed by 45 cycles of two-step PCR consisting of 95 °C for 15 s and 62 °C for 60 s. Samples with a cycle threshold (Ct) value of 38 or less were considered positive, calculated by averaging triplicates for each sample.

Previous literature suggests that this commercially available assay cross-reacts with other *Mycobacterium* species [61]. Due to the suggested possible cross-reactivity between the commercial extraction assay and other *Mycobacterium* species, samples were tested using three previously published gene targets in a multiplex format validated at WVDL according to AAVLD guidelines. The assay has linearity and efficiency of 0.997 and 100%, respectively. The diagnostic sensitivity is 92.5% (95% confidence interval, 83% to 97.5%) and diagnostic specificity is 100% (95% confidence interval, 98.9% to 100%) when compared to the commercially available assay. Briefly, a 1-step reverse transcription assay followed by probe-based amplification was performed for 40 cycles in a real-time thermocycler instrument (ABI 750, Thermo Fisher Scientific, Waltham, MA, USA). A positive amplification control, an extraction control, and a negative control were performed with each assay. Cycle threshold (C_T_) levels were determined, where values ≤36 were considered positive results and values ≥37 but <40 were considered inconclusive results. C_T_ values ≥ 40 were considered to be below the level of detection.

### 4.6. Acid-Fast Bacilli Evaluation

Tissue sections belonging to the small intestine and mediastinal and jejunal lymph nodes were used to evaluate acid-fast bacilli (AFB) once fixed and stained using Ziehl-Neelsen staining. Evaluation of AFB in tissues was conducted according to the same protocol published previously, which included all FFPE tissues [62]. Briefly, the presence of acid-fast rod-shaped bacteria is graded as: 0—not present/not seen; 1—present, relatively low numbers; 2—present, moderate numbers; 3—present, numerous. The four samples from WVDL were evaluated by Dr. Philip N. Bochsler, DVM, PhD, DACVP, using the criteria above. 

### 4.7. Case-Control Criteria

For the FFPE dataset alone, cases were defined as tissue qPCR/culture-positive or evidence of AFB in tissues. In contrast, controls were defined as tissue qPCR and culture-negative without evidence of AFB in tissues. Cases were considered infected and controls were considered not infected based upon the terminology proposed for JD literature previously [4]. 

### 4.8. SNP Genotyping and Quality Control

Samples were genotyped using Illumina’s OvineSNP50 BeadChip, which contains 53,516 SNPs aligned to the publicly available Oar_v4.0 *Ovis aries* genome assembly submitted by the International Sheep Genome Consortium (ISGC). FFPE samples underwent restoration via Illumina’s FFPE Restoration kit prior to genotyping. A starting genomic DNA amount of 100 ng per sample was used and underwent multiple incubation steps using proprietary Illumina buffers and enzymatic steps to identify the SNP signatures of each individual. All Illumina microarrays were run using the Illumina iScan technology at the Mammalian Genotyping Core at the University of North Carolina at Chapel Hill by the same technologist. 

SNP pruning and QC were conducted using both Illumina’s GenomeStudio software v2.0.5 and PLINK v1.90 Samples with call rates <0.8 were removed, and SNPs with call rates <0.9 were also removed. SNPs with MAF <0.01 were filtered out for rare variants. 

### 4.9. Case-Control Association Analysis via GEMMA

To identify SNPs significantly associated with cases in this study, genome-wide efficient mixed-model association (GEMMA) 0.98.1 was utilized and aided in accounting for relatedness and controlling population stratification for association analysis using binary outcomes (case-control) in a linear mixed model (LMM) logistic regression [63]. Breed and age were used as co-variates, and the likelihood-ratio test was used to calculate *p*-values. Multiple testing corrections were conducted using the protocols outlined in (32) in Rstudio using the poolr package [64]. This correction accounts for multiple testing corrections while being less stringent compared to the Bonferroni or Sidak corrections for genome-wide significance [64].

GEMMA analysis for a univariate linear mixed model followed the following:

y = Wα + xβ + u + ε; u∼MVN_n_ (0, λτ^−1^K), ε∼MVN_n_ (0, τ^−1^I_n_)

where y is a n-vector of binary disease labels (1 = cases, 0 = control) for n individuals. W equals (w1, …, wc) is an nxc matrix of covariates (breed and age), α is a c-vector of the corresponding coefficients including the intercept, X is an n-vector of marker genotypes, β is the effect size of the marker, u is an n-vector of random effects, ε is an n-vector of errors, τ^−1^ is the variance of the residual errors, λ is the ratio between the two variance components, K is a known n x n relatedness matrix, and I_n_ is an n × n identity matrix. 

GEMMA tests the alternative hypothesis H_1_: β ≠ 0 against the null hypothesis H_0_: β = 0 for each SNP using the likelihood ratio test. GEMMA accounted for population differences using the random effect u, specifically the K matrix.

SNPs with missing genotypes were imputed using Beagle 5.4 for several datasets without reference information [65]. SNPs significantly associated with cases vs. controls in this analysis are those above the genome-wide significance threshold calculated. Manhattan plots show the significant SNPs above the line, which marks the genome-wide significance threshold. The Rstudio package qqman was used to generate the Manhattan plot and QQ plots. 

### 4.10. Gene Identification from Significant SNPs

The location of each significant SNP in relation to surrounding genes was evaluated using the NCBI Genome Data Viewer on the Oar_v4.0 *Ovis aries* genome assembly. Genes harboring SNPs and genes in a 1 Mb window (500 Kb up-and downstream) of a significant SNP were identified and further evaluated for relationship with MAP infection. Gene symbol, name, chromosome location, and associated SNP were recorded and used for downstream pathway analysis. 

### 4.11. Gene Ontology and Pathway Analysis

Genes found in relation to the significant SNPs were imputed into PANTHER 17.0 for a Gene Ontology (GO) and pathway analysis using human-derived data regarding functional classes and related pathways [66,67]. PANTHER GO for biological processes was a run and pie chart containing the distribution of processes generated. PANTHER pathway analysis was conducted and also generated a pie chart of the pathway distributions for the genes found by PANTHER. 

To further investigate species-specific pathways, genes identified in the PANTHER GO and pathway analyses, as well as any genes containing SNPs, were subjected to KEGG pathway analysis. Individual genes were searched for ovine-specific pathways; results recorded pathways harboring the most genes, and pathways related to host response to MAP infection were identified. Total pathways and the number of genes per pathway were identified. 

## 5. Conclusions

The mechanisms underlying natural host resistance/susceptibility to MAP infection are still unknown; however, studies over the past two decades utilizing host genetics and gene expression studies have shed light on common pathways found across species. These pathways and genes have most commonly been related to functions of recognition and the initiation of downstream pro-inflammatory signaling. Identifying these genes and pathways across studies alludes to changes in host-pathogen interactions that occur early in infection being vital and setting the stage for the remaining infection. Our work adds to the limited GWAS studies investigating host resistance/susceptibility to ovine Johne’s disease (OJD) and provides a framework for future successful use of FFPE-derived DNA GWAS studies in livestock species. 

The work presented here adds to the current knowledge base of immune system pathways involved in susceptibility/resistance to MAP, validating commonly conserved pathways involved in innate immunity. We also identified pathways involved in the adaptive immune system, including both B and T cell receptor signaling, through a single candidate gene, *IKBKB*. Future work should build upon the results presented here and include increased numbers of cases and controls meeting strict case-control definitions. Future meta-analysis building upon this data could aid in identifying and validating the SNPs, genes, and pathways identified here and provide insight into breed specific and mixed-breed analysis studies. Particularly, the utilization of FFPE-derived DNA in this retrospective study provides evidence for these samples to be shared and utilized along with archived clinical or diagnostic data to enrich similar analyses in the future. 

## Figures and Tables

**Table 1:** Case Control Definition Criteria.

Case	Control
Tissue qPCR or culture-positive, or acid-fast bacilli in tissues	Tissue qPCR and culture negative, no acid-fast bacilli in tissues
N = 9	N = 25

**Table 2:** FFPE GEMMA SNP Results.

SNP ID	Chromosome	Position	*p*-Value	Allele	NCBI SNP ID
DU236737_223.1	1	275076998	2.52 × 10^−8^	C/T	rs401362015
OAR1_124914407.1	1	115770091	1.48 × 10^−8^	T/C	rs407060336
s61231.1	1	149950550	1.01 × 10^−8^	T/C	rs410166885
s71828.1	3	39133628	6.12 × 10^−7^	C/T	rs428083866
OAR3_180416904.1	3	167767251	3.79 × 10^−7^	T/C	rs401844951
s52576.1	3	165574034	1.26 × 10^−9^	A/G	rs399773060
s49867.1	4	46552546	5.04 × 10^−9^	C/A	rs406625389
DU442221_261.1	24	33324074	1.92 × 10^−11^	G/A	rs55627888
s13427.1	26	30765074	4.70 × 10^−10^	A/C	rs415353783
OAR26_40962143.1	26	36010402	4.25 × 10^−7^	A/C	rs399723913

*p* < 1 × 10^−6^ SNP N = 10 SNP ID from the Illumina Ovine SNP50 BeadChip assay ID, chromosome (OAR), and position based upon the Oar_v4.0 *Ovis aries* genome assembly where alleles and SNP rs numbers were recorded from the NCBI Genome Browser.

**Table 3:** FFPE GEMMA Gene Results List *p* < 1 × 10^−6^ N = 45 genes found within a 1 Mb window using the NCBI Genome Browser *OarV4.0* genome assembly.

NCBI SNP ID	Chromosome	Position	Gene Symbol within 1 Mb
rs401362015	1	275076998	RAB5A-within gene intronic
rs407060336	1	115770091	TMCO1-within gene
rs410166885	1	149950550	N/A
rs428083866	3	39133628	ANTXR1-within gene
rs401844951	3	167767251	ANKS1B—within gene
rs399773060	3	165574034	CFAP54—within gene
rs406625389	4	46552546 6	LOC101104484, PUS7, RINT1, ATXN7L1, SRPK2, KMT2E
rs55627888	24	33324074 18	LOC105604867, TMEM270, METTL27, CLDN4, CLDN3, ABHD11, STX1A, BUD23, VPS37D, MLXIPL, TBL2, BCL7B, BAZ1B, FZD9, FKBP6, TRIM50, NSUN5, POM121C
rs415353783	26	30765074	KCNU1—within gene
rs399723913	26	36010402 15	THAP1, RNF170, HOOK3, FNTA, POMK, HGSNAT, INTS10, CHRNA6, CHRNB3, SMIM19, SLC20A2, VDAC3, POLB, IKBKB, PLAT

**Table 4:** Possible Candidate Genes identified, associated SNPs, and *p*-values for genes found to be physiologically relevant and function in immunity.

NCBI SNP ID	Chromosome	Position	*p*-Value	Gene Symbol
rs55627888	24	33324074	1.92 × 10^−11^	FZD9 310,853 bp upstream
rs401362015	1	275076998	2.52 × 10^−8^	RAB5A (within gene-intronic)
rs428083866	3	39133628	6.12 × 10^−7^	ANTXR1 (within gene)
rs399723913	26	36010402	4.25 × 10^−7^	IKBKB 398,692 bp downstream

**Table 5:** Pathways functioning in innate immunity and immune signaling N = 9 pathways with primary function to innate immunity, recognition of bacteria, and subsequent inflammatory signaling in order to mount an appropriate response to MAP infection. Bolded genes are part of the four target potential candidate genes.

KEGG Pathway Name	Genes in Pathway
NOD-like receptor signaling pathway	ANTXR1, IKBKB, VDAC3
Cell adhesion molecules	CLDN4, CLDN3
Phagosome	RAB5A
Tuberculosis	RAB5A
NF-kappa B signaling pathway	IKBKB
Toll-like receptor signaling pathway	IKBKB
TNF signaling pathway	IKBKB
Wnt signaling pathway	FZD9
IL-17 signaling pathway	IKBKB

**Table 6:** Pathways Functioning in the adaptive immune system (N = 4).

KEGG Pathway Name	Genes in Pathway
Th1 and Th2 cell differentiation	IKBKB
Th17 cell differentiation	IKBKB
T cell receptor signaling pathway	IKBKB
B cell receptor signaling pathway	IKBKB

## Data Availability

The datasets presented in this article at this time are not readily available due to the data being part of an ongoing genetic analysis study. Data is available upon request from the corresponding authors.

## Supplementary Materials

The following supporting information can be downloaded at: https://www.mdpi.com/article/10.3390/ijms25147748/s1.
