# Supplementary material for: Retrospective Single Nucleotide Polymorphism Analysis of Host Resistance and Susceptibility to Ovine Johne’s Disease Using Restored FFPE DNA
Source: Int J Mol Sci. 2024 Jul 15;25(14):7748. doi: 10.3390/ijms25147748 (PMC11276633; doi:10.3390/ijms25147748)
Supplement: Supplementary file 1 [file ijms-25-07748-s001.zip › S1 IJMS 2024.docx]

**Supplementary Table 1: FFPE Case-Control Tables**

| **Sample ID** | **Origin Flock** | **Breed** | **Age at Necropsy (years)** | **Gender (M/F)** | **Fecal (q)PCR** | **Tissue (q)PCR** | **Acid-Fast Histopathology** |
| --- | --- | --- | --- | --- | --- | --- | --- |
| JD184 | M | Kattapakkam Red | 7 | F | POS | POS | 0 |
| JD692 | M | Kattapakkam Red | 3 | F | POS | POS | 0 |
| JD738 | M | Kattapakkam Red | 3 | F | POS | POS | 1 (SI) |
| OKST 2 | OKST | N/A | 2 | F | POS | N/A | + ILIUM |
| JD656 | M | Kattapakkam Red | 3 | F | POS | NEG | 2 (SI) |
| TR-15111 | TR | Royal White x White Dorper | 4 | F | POS | POS | 2 (SI) 1(LI) |
| TR-1741 | TR | Royal White | 2 | F | POS | POS | FEW |
| TR-J24 | TR | Royal White x White Dorper | 2 | F | POS | POS | 0 |
| TR-4106 | TR | White Dorper x Dorcet | 5 | F | POS | POS | 3 (SI) |

**Table 1a Cases:** List of cases (N=9) with positive fecal qPCR results and evidence of tissue infection.

| **Sample ID** | **Origin Flock** | **Breed** | **Age at Necropsy (years)** | **Gender (M/F)** | **Fecal (q)PCR** | **Tissue (q)PCR** | **Acid-Fast Histopathology** |
| --- | --- | --- | --- | --- | --- | --- | --- |
| C257 (C1) | M | Kattapakkam Red | 6 | F | POS | NEG | 0 |
| C168 | M | Kattapakkam Red | 7 | F | POS | NEG | 0 |
| C374 | M | Kattapakkam Red | 5 | F | POS | NEG | 0 |
| C495 | M | Kattapakkam Red | 2 | F | POS | NEG | 0 |
| C424 | M | Kattapakkam Red | 5 | F | POS | NEG | 0 |
| C699 | M | Kattapakkam Red | 2 | M | POS | NEG | 0 |
| JD254 | M | Kattapakkam Red | 6 | F | POS | NEG | 0 |
| JD522 | M | Kattapakkam Red | 4 | F | POS | NEG | 0 |
| JD660 | M | Kattapakkam Red | 2 | F | POS | NEG | 0 |
| JD803 | M | Kattapakkam Red | 2 | M | POS | NEG | 0 |
| MJD347 | M | Kattapakkam Red | 6 | F | POS | NEG | 0 |
| MJD533 | M | Kattapakkam Red | 4 | F | POS | NEG | 0 |
| MJD637 | M | Kattapakkam Red | 3 | F | POS | NEG | 0 |
| MJD663 | M | Kattapakkam Red | 2 | M | POS | NEG | 0 |
| MJD826 | M | Kattapakkam Red | 2 | M | POS | NEG | 0 |
| MJD890 | M | Kattapakkam Red | 2 | M | POS | NEG | 0 |
| MJD 790 | M | Kattapakkam Red | 2 | M | POS | NEG | 0 |
| MJD 741 | M | Kattapakkam Red | 3 | F | POS | NEG | 0 |
| MJD 653 | M | Kattapakkam Red | 5 | F | POS | NEG | 0 |
| M375 | M | Kattapakkam Red | 5 | F | POS | NEG | 0 |
| M437 | M | Kattapakkam Red | 2 | F | POS | NEG | 0 |
| M510 | M | Kattapakkam Red | 3 | F | POS | NEG | 0 |
| M724 | M | Kattapakkam Red | 2 | F | POS | NEG | 0 |
| M412 | M | Kattapakkam Red | 5 | F | POS | NEG | 0 |
| M616 | M | Kattapakkam Red | 3 | F | POS | NEG | 0 |

**Table 1b Controls:** List of controls (N=25) fecal qPCR positive but without evidence of tissue infection.
