# Supplementary material for: Retrospective Single Nucleotide Polymorphism Analysis of Host Resistance and Susceptibility to Ovine Johne’s Disease Using Restored FFPE DNA
Source: Int J Mol Sci. 2024 Jul 15;25(14):7748. doi: 10.3390/ijms25147748 (PMC11276633; doi:10.3390/ijms25147748)
Supplement: Supplementary file 1 [file ijms-25-07748-s001.zip › S2 IJMS.docx]

**Supplementary Table 2: FFPE Gene Functions**

**S2 Table FFPE Gene Functions:** Gene functions associated with identified significant SNPs from GWAS. All genes and functions were adapted from NCBI Genome.

| **Chromosome OAR** | **NCBI SNP ID** | **Gene Symbol** | **Gene Name and Function** | **SNP location in gene** |
| --- | --- | --- | --- | --- |
| 1 | rs401362015 | RAB5A | RAB5A member RAS oncogene family  Enables GDP, GTP, and GTPase activity. (wnt signaling, autophagosome) | Within gene |
| 1 | rs407060336 | TMCO1 | Transmembrane and coiled-coil domains 1  Encoded transmembrane protein, involved in calcium channel activity. | Within gene |
| 1 | rs410166885 | N/A | N/A | N/A |
| 3 | rs428083866 | ANTXR1 | ANTXR cell adhesion molecule 1  Encodes type I transmembrane protein, docking receptor for *Bacillus anthracis* toxin. | Within gene |
| 3 | rs401844951 | ANKS1B | Ankyrin repeat and sterile alpha domain containing 1B  Envolved in brain development and pathogenesis of Alzheimer's disease. | Within gene |
| 3 | rs399773060 | CFAP54 | Cilia and flagella associated protein 54  Predicted involvement in cilium movement involved in cell motility. | Within gene |
| 4 | rs406625389 | LOC101104484    PUS7    RINT1    ATXN7L1  SRPK2    KMT2E | Nucleotide triphosphate diphosphatase NUT15-like  Pseudouridine synthase 7  Enables enzyme binding activity and pseudouridine synthesis.  RAD50 interactor 1  Encoded protein involved in regulation of cell cycle progression, role in trafficking cellular cargo from endosome.  Ataxin 7 like 1  SRSF protein kinase 2  Involved in nucleic acid metabolism, regulation of viral replication, and involved in innate immune response.  Lysine methyltransferase 2E  Member of the myeloid/lymphoid or mixed-lineage leukemia (MLL) family of proteins, overexpression inhibits cell cycle progression. | 10,635bp upstream    63,974bp upstream    108,255bp upstream    282,590bp  upstream  140,876bp  downstream    306,504bp  downstream |
| 24 | rs55627888 | TMEM270  METTL27  CLDN4    CLDN3    ABHD11    STX1A    BUD23    VPS37D    MLXIPL    TBL2    BCL7B    BAZ1B    FZD9    FKBP6    TRIM50    NSUN5    POM121C | Transmembrane protein 270  Methyltransferase like 27  Claudin 4  Encodes a member of the Claudin family of integral membrane proteins within epithelial cell tight junctions. High affinity receptor for *Clostridium perfringens* enterotoxin  Claudin 3  Member of the Claudin family of integral membrane proteins, a component of tight junction strands. Involved in host response to gram positive bacteria.  Abhydrolase domain containing 11  Syntaxin 1A  Encodes member of syntaxin superfamily of nervous system-system specific proteins. Key molecule in ion channel regulation and synaptic exocytosis.  BUD23 rRNA methyltransferase and ribosome maturation factor  Encoded protein suggested to be involved in DNA methylation.  VPS37D subunit ESCRT-I  Predicted to be involved in protein targeting to vacuole, and ubiquitin–dependent protein catabolic processes.  MLX interacting protein like  Codes for a transcription factor, promotes triglyceride synthesis genes.  Transducin beta like 2  Encodes member of the beta-transduction protein family, involved in regulation of intracellular signaling.  BAF chromatin remodeling complex subunit BCL7B  Gene encodes member of the BCL7 family of proteins, involved in **wnt signaling pathway?**  Bromodomain adjacent to zinc finger domain 1B  Encodes member of bromodomain protein family, involved in chromatin-dependent regulation of transcription.  Frizzled class receptor 9  Encodes for a 7-transmembrane protein that function as receptors for Wnt signaling proteins.  FKBP prolyl isomerase family member 6  Encodes a cis-trans peptidyl-prolyl isomerase proposed to be involved in immunoregulation and basic cellular processes.  E3 ubiquitin-protein ligase TRIM50  NOP2/Sun RNA methyltransferase 5  Member of a conserved protein family thought to function as methyltransferases.  POM121 transmembrane nucleoporin C  Predicted to encode protein involved in nuclear localization sequence binding activity.  E3 ubiquitin-protein ligase TRIM50  NOP2/Sun RNA methyltransferase 5  Member of a conserved protein family thought to function as methyltransferases.  POM121 transmembrane nucleoporin C  Predicted to encode protein involved in nuclear localization sequence binding activity. | 23,765bp upstream  53,426bp upstream  57,199bp  upstream    87,600 bp upstream    112,155bp upstream    136,293bp upstream    147,986bp upstream    164,464bp upstream    203,427bp upstream    225,892 bp upstream    241,257bp upstream    279,959bp upstream    310,853bp upstream    372,676bp upstream    392,672bp upstream  401,307bp upstream    418,031bp upstream |
| 26 |  | KCNU1 | Potassium calcium-activated channel subfamily U member 1  Encodes a voltage-gated ion channel, involved in outward potassium ion flow during plasma membrane hyperpolarization in sperm. | Within gene |
| 26 |  | THAP1    RNF170    HOOK3    FNTA    POMK    HGSNAT    INTS10    CHRNA6    CHRNB3    SMIM19    SLC20A2    VDAC3    POLB    IKBKB    PLAT | THAP domain containing 1  Encoded protein contains a conserved THAP DNA-binding domain, acts as a proapoptotic factor.  Ring finger protein 170  Encoded protein functions as an E3 ubiquitin ligase.  Hook microtubule tethering protein 3  Encodes a cytosolic coiled-coil protein involved in microtubule mediated binding to organelles.  Farnesyltransferase, CAAX box, alpha  Gene encodes alpha subunit of a prenyltransferase group of transferases.  Protein O-mannose kinase  Encodes protein involved in formation of transmembrane linkages between extracellular matrix and exoskeleton, O-linked carbohydrate unit used by some pathogens for host entry.  Heparan-alpha-glucosaminidine N-acetyltransferase  Encodes a lysosomal acetyltransferase involved in lysosomal degradation of heparin sulfate.  Integrator complex subunit 10  Encodes subunit of the Integrator complex involved in 3-prime end processing of small nuclear RNAs.  Cholinergic receptor nicotinic alpha 6 subunit  Encodes an alpha subunit of neuronal nicotinic acetylcholine receptors that function as ion channels.  Cholinergic receptor nicotinic beta 3 subunit  Encodes member of the nicotinic acetylcholine receptors (nAChRs) that function as ligand-gated ion channels that mediate fast signal transmission at synapses.  Small integral membrane protein 19  Solute carrier family 20 member 2  Encodes a type 3 sodium-dependent phosphate symporter, involved in phosphate homeostasis and susceptibility to viral infection as a gamma-retroviral receptor.  Voltage dependent anion channel 3  Encodes a voltage-dependent anion channel (VDAC), involved in mitochondrial permeability in apoptosis.  DNA polymerase beta  Encodes a DNA polymerase involved in base excision and repair.  Inhibitor of nuclear factor kappa B kinase subunit beta  Encoded protein phosphorylates the inhibitor in the inhibitor/NF-kappa-B complex resulting in activation of NF-kappa-B.  Plasminogen activator tissue type  Encodes a secreted serine protease responsible for conversion of plasminogen to plasmin. | 18,680bp upstream    48,451bp upstream    109,402bp upstream    189,976bp upstream    214,256bp upstream    248,466bp upstream    285,309bp upstream    22,747bp downstream    55,225bp  downstream    171,381bp downstream  233,424bp downstream    299,120bp downstream    340,954bp downstream    398,692bp downstream    486,876bp downstream |
