# Supplementary material for: Retrospective Single Nucleotide Polymorphism Analysis of Host Resistance and Susceptibility to Ovine Johne’s Disease Using Restored FFPE DNA
Source: Int J Mol Sci. 2024 Jul 15;25(14):7748. doi: 10.3390/ijms25147748 (PMC11276633; doi:10.3390/ijms25147748)
Supplement: Supplementary file 1 [file ijms-25-07748-s001.zip › S3 IJMS.docx]

**Supplementary Table 3: Full List of KEGG Pathways and Genes**

**Table S3:** (N = 74) total Ovine KEGG Pathways Identified using 16 physiologically relevant genes based upon known MAP-host pathophysiology.

| **Pathway Number** | **KEGG Pathway ID** | **Pathway Name** | **Genes** |
| --- | --- | --- | --- |
|  |  |  |  |
| 1 | **oas04014** | Ras signaling pathway | RAB5A |
|  |  |  | IKBKB |
| 2 | **oas04144** | Endocytosis | RAB5A |
|  |  |  | VPS37D |
| 3 | oas04145 | Phagosome | RAB5A |
|  |  |  |  |
| 4 | oas04962 | Vasopressin-regulated water reabsorption | RAB5A |
|  |  |  |  |
| 5 | oas05014 | Amyotrophic lateral sclerosis | RAB5A |
|  |  |  |  |
| 6 | **oas05022** | Pathways of neurodegeneration - multiple diseases | RAB5A |
|  |  |  | STX1A |
|  |  |  | FZD9 |
|  |  |  | VDAC3 |
| 7 | **oas05132** | Salmonella infection | RAB5A |
|  |  |  | IKBKB |
| 8 | oas05146 | Amoebiasis | RAB5A |
|  |  |  |  |
| 9 | oas05152 | Tuberculosis | RAB5A |
|  |  |  |  |
| 10 | **oas04621** | NOD-like receptor signaling pathway | ANTXR1 |
|  |  |  | IKBKB |
|  |  |  | VDAC3 |
| 11 | **oas04022** | cGMP-PKG signaling pathway | KCNU1 |
|  |  |  | VDAC3 |
| 12 | oas04270 | Vascular smooth muscle contraction | KCNU1 |
|  |  |  |  |
| 13 | **oas04911** | Insulin secretion | KCNU1 |
|  |  |  | STX1A |
| 14 | oas01523 | Antifolate resistance | IKBKB |
|  |  |  |  |
| 15 | oas04010 | MAPK signaling pathway | IKBKB |
|  |  |  |  |
| 16 | oas04062 | Chemokine signaling pathway | IKBKB |
|  |  |  |  |
| 17 | oas04064 | NF-kappa B signaling pathway | IKBKB |
|  |  |  |  |
| 18 | oas04068 | FoxO signaling pathway | IKBKB |
|  |  |  |  |
| 19 | **oas04150** | mTOR signaling pathway | IKBKB |
|  |  |  | FZD9 |
| 20 | oas04151 | PI3K-Akt signaling pathway | IKBKB |
|  |  |  |  |
| 21 | oas04210 | Apoptosis | IKBKB |
|  |  |  |  |
| 22 | oas04380 | Osteoclast differentiation | IKBKB |
|  |  |  |  |
| 23 | oas04620 | Toll-like receptor signaling pathway | IKBKB |
|  |  |  |  |
| 24 | oas04622 | RIG-I-like receptor signaling pathway | IKBKB |
|  |  |  |  |
| 25 | oas04623 | Cytosolic DNA-sensing pathway | IKBKB |
|  |  |  |  |
| 26 | oas04625 | C-type lectin receptor signaling pathway | IKBKB |
|  |  |  |  |
| 27 | oas04657 | IL-17 signaling pathway | IKBKB |
|  |  |  |  |
| 28 | oas04658 | Th1 and Th2 cell differentiation | IKBKB |
|  |  |  |  |
| 29 | oas04659 | Th17 cell differentiation | IKBKB |
|  |  |  |  |
| 30 | oas04660 | T cell receptor signaling pathway | IKBKB |
|  |  |  |  |
| 31 | oas04662 | B cell receptor signaling pathway | IKBKB |
|  |  |  |  |
| 32 | oas04668 | TNF signaling pathway | IKBKB |
|  |  |  |  |
| 33 | oas04722 | Neurotrophin signaling pathway | IKBKB |
|  |  |  |  |
| 34 | oas04910 | Insulin signaling pathway | IKBKB |
|  |  |  |  |
| 35 | oas04920 | Adipocytokine signaling pathway | IKBKB |
|  |  |  |  |
| 36 | oas04931 | Insulin resistance | IKBKB |
|  |  |  |  |
| 37 | **oas05010** | Alzheimer disease | IKBKB |
|  |  |  | VDAC3 |
|  |  |  | FZD9 |
|  |  |  |  |
| 38 | oas05135 | Yersinia infection | IKBKB |
|  |  |  |  |
| 39 | oas05145 | Toxoplasmosis | IKBKB |
|  |  |  |  |
| 40 | **oas05160** | Hepatitis C | IKBKB |
|  |  |  | CLDN4 |
|  |  |  | CLDN3 |
| 41 | **oas05161** | Hepatitis B | IKBKB |
|  |  |  | VDAC3 |
| 42 | oas05164 | Influenza A | IKBKB |
|  |  |  |  |
| 43 | oas05167 | Kaposi sarcoma-associated herpesvirus infection | IKBKB |
|  |  |  |  |
| 44 | oas05171 | Coronavirus disease | IKBKB |
|  |  |  |  |
| 45 | **oas05200** | Pathways in cancer | IKBKB |
|  |  |  | FZD9 |
| 46 | oas05220 | Chronic myeloid leukemia | IKBKB |
|  |  |  |  |
| 47 | oas05235 | PD-L1 expression and PD-1 checkpoint pathway in cancer | IKBKB |
|  |  |  |  |
| 48 | oas05417 | Lipid and atherosclerosis | IKBKB |
|  |  |  |  |
| 49 | oas04130 | SNARE interactions in vesicular transport | STX1A |
|  |  |  |  |
| 50 | oas04721 | Synaptic vesicle cycle | STX1A |
|  |  |  |  |
| 51 | **oas05016** | Huntington disease | STX1A |
|  |  |  | VDAC3 |
| 52 | **oas04080** | Neuroactive ligand-receptor interaction | CHRNA6 |
|  |  |  | CHRNB3 |
| 53 | oas04725 | Cholinergic synapse | CHRNA6 |
|  |  |  |  |
| 54 | oas04310 | Wnt signaling pathway | FZD9 |
|  |  |  |  |
| 55 | oas04390 | Hippo signaling pathway | FZD9 |
|  |  |  |  |
| 56 | oas04550 | Signaling pathways regulating pluripotency of stem cells | FZD9 |
|  |  |  |  |
| 57 | oas04934 | Cushing syndrome | FZD9 |
|  |  |  |  |
| 58 | oas05205 | Proteoglycans in cancer | FZD9 |
|  |  |  |  |
| 59 | oas05225 | Hepatocellular carcinoma | FZD9 |
|  |  |  |  |
| 60 | oas05226 | Gastric cancer | FZD9 |
|  |  |  |  |
| 61 | oas04371 | Apelin signaling pathway | PLAT |
|  |  |  |  |
| 62 | oas04610 | Complement and coagulation cascades | PLAT |
|  |  |  |  |
| 63 | oas05202 | Transcriptional misregulation in cancer | PLAT |
|  |  |  |  |
| 64 | **oas04514** | Cell adhesion molecules | CLDN4 |
|  |  |  | CLDN3 |
|  |  |  |  |
| 65 | **oas04530** | Tight junction | CLDN4 |
|  |  |  | CLDN3 |
| 66 | **oas04670** | Leukocyte transendothelial migration | CLDN4 |
|  |  |  | CLDN3 |
| 67 | oas04020 | Calcium signaling pathway | VDAC3 |
|  |  |  |  |
| 68 | oas04216 | Ferroptosis | VDAC3 |
|  |  |  |  |
| 69 | oas04217 | Necroptosis | VDAC3 |
|  |  |  |  |
| 70 | oas04218 | Cellular senescence | VDAC3 |
|  |  |  |  |
| 71 | oas04613 | Neutrophil extracellular trap formation | VDAC3 |
|  |  |  |  |
| 72 | oas04979 | Cholesterol metabolism | VDAC3 |
|  |  |  |  |
| 73 | oas05020 | Prion disease | VDAC3 |
|  |  |  |  |
| 74 | oas05203 | Viral carcinogenesis | VDAC3 |
