# Supplementary material for: Retrospective Single Nucleotide Polymorphism Analysis of Host Resistance and Susceptibility to Ovine Johne’s Disease Using Restored FFPE DNA
Source: Int J Mol Sci. 2024 Jul 15;25(14):7748. doi: 10.3390/ijms25147748 (PMC11276633; doi:10.3390/ijms25147748)
Supplement: Supplementary file 1 [file ijms-25-07748-s001.zip › S4 IJMS.docx]

**Supplementary Table S4: MAP Associated KEGG Pathways and Genes**

**Table S4:** List of pathways and associated genes most physiologically related to MAP infection N = 19

| **KEGG Pathway ID** | **Pathway Name** | **Genes in Pathway** | **NCBI SNP ID** | **Chromosome** |
| --- | --- | --- | --- | --- |
| oas04621 | NOD-like receptor signaling pathway | **ANTXR1**  **IKBKB**  VDAC3 | rs428083866  rs399723913  rs399723913 | 3  26  26 |
| oas04514 | Cell adhesion molecules | CLDN4  CLDN3 | rs55627888  rs55627888 | 24  24 |
| oas04145 | Phagosome | **RAB5A** | rs401362015 | 1 |
| oas05152 | Tuberculosis | **RAB5A** | s401362015 | 1 |
| oas04064 | NF-kappa B signaling pathway | **IKBKB** | rs399723913 | 26 |
| oas04620 | Toll-like receptor signaling pathway | **IKBKB** | rs399723913 | 26 |
| oas04530 | TNF signaling pathway | **IKBKB** | rs399723913 | 26 |
| oas04310 | Wnt signaling pathway | **FZD9** | rs55627888 | 24 |
| oas04657 | IL-17 signaling pathway | **IKBKB** | rs399723913 | 26 |
| oas04658 | Th1 and Th2 cell differentiation | **IKBKB** | rs399723913 | 26 |
| oas04659 | Th17 cell differentiation | **IKBKB** | rs399723913 | 26 |
| oas04660 | T cell receptor signaling pathway | **IKBKB** | rs399723913 | 26 |
| oas04662 | B cell receptor signaling pathway | **IKBKB** | rs399723913 | 26 |
| oas04014 | Ras signaling pathway | **RAB5A**  **IKBKB** | rs401362015  rs399723913 | 1  26 |
| oas04144 | Endocytosis | **RAB5A**  VPS37D | rs401362015 | 1 |
| oas05132 | Salmonella infection | **RAB5A**  **IKBKB** | s401362015  rs399723913 | 1  26 |
| oas04150 | mTOR signaling pathway | **IKBKB**  **FZD9** | rs399723913  rs55627888 | 26  24 |
| oas04530 | Tight junction | CLDN4  CLDN3 | rs55627888  rs55627888 | 24  24 |
| oas04670 | Leukocyte transendothelial migration | CLDN4  CLDN3 | rs55627888  rs55627888 | 24  24 |
