# Supplementary material for: Retrospective Single Nucleotide Polymorphism Analysis of Host Resistance and Susceptibility to Ovine Johne’s Disease Using Restored FFPE DNA
Source: Int J Mol Sci. 2024 Jul 15;25(14):7748. doi: 10.3390/ijms25147748 (PMC11276633; doi:10.3390/ijms25147748)
Supplement: Supplementary file 1 [file ijms-25-07748-s001.zip › S4 IJMS.pdf]

Supplementary Table S4: MAP Associated KEGG Pathways and Genes

Table S4: List of pathways and associated genes most physiologically related to MAP infection N = 19

KEGG Pathway ID	Pathway Name	Genes in Pathway	NCBI SNP ID	Chromosome
oas04621	NOD-like receptor signaling pathway	**ANTXR1**	rs428083866	3
		**IKBKB**	rs399723913	26
		VDAC3	rs399723913	26
oas04514	Cell adhesion molecules	CLDN4	rs55627888	24
		CLDN3	rs55627888	24
oas04145	Phagosome	**RAB5A**	rs401362015	1
oas05152	Tuberculosis	**RAB5A**	s401362015	1
oas04064	NF-kappa B signaling pathway	**IKBKB**	rs399723913	26
oas04620	Toll-like receptor signaling pathway	**IKBKB**	rs399723913	26
oas04530	TNF signaling pathway	**IKBKB**	rs399723913	26
oas04310	Wnt signaling pathway	**FZD9**	rs55627888	24
oas04657	IL-17 signaling pathway	**IKBKB**	rs399723913	26
oas04658	Th1 and Th2 cell differentiation	**IKBKB**	rs399723913	26
oas04659	Th17 cell differentiation	**IKBKB**	rs399723913	26
oas04660	T cell receptor signaling pathway	**IKBKB**	rs399723913	26
oas04662	B cell receptor signaling pathway	**IKBKB**	rs399723913	26
oas04014	Ras signaling pathway	**RAB5A**	rs401362015	1
		**IKBKB**	rs399723913	26
oas04144	Endocytosis	**RAB5A**	rs401362015	1
		VPS37D		

oas05132	Salmonella infection	**RAB5A**	s401362015	1
		**IKBKB**	rs399723913	26
oas04150	mTOR signaling pathway	**IKBKB**	rs399723913	26
		**FZD9**	rs55627888	24
oas04530	Tight junction	CLDN4	rs55627888	24
		CLDN3	rs55627888	24
oas04670	Leukocyte transendothelial migration	CLDN4	rs55627888	24
		CLDN3	rs55627888	24
