# Supplementary material for: Retrospective Single Nucleotide Polymorphism Analysis of Host Resistance and Susceptibility to Ovine Johne’s Disease Using Restored FFPE DNA
Source: Int J Mol Sci. 2024 Jul 15;25(14):7748. doi: 10.3390/ijms25147748 (PMC11276633; doi:10.3390/ijms25147748)
Supplement: Supplementary file 1 [file ijms-25-07748-s001.zip › S1 IJMS 2024.pdf]

Supplementary Table S1: FFPE Case-Control Tables

Sample ID	Origin Flock	Breed	Age at Necropsy (years)	Gender (M/F)	Fecal (q)PCR	Tissue (q)PCR	Acid-Fast Histopathology
JD184	M	Kattapakkam Red	7	F	POS	POS	0
JD692	M	Kattapakkam Red	3	F	POS	POS	0
JD738	M	Kattapakkam Red	3	F	POS	POS	1 (SI)
OKST 2	OKST	N/A	2	F	POS	N/A	+ ILIUM
JD656	M	Kattapakkam Red	3	F	POS	NEG	2 (SI)
TR-15111	TR	Royal White x White Dorper	4	F	POS	POS	2 (SI) 1(LI)
TR-1741	TR	Royal White	2	F	POS	POS	FEW
TR-J24	TR	Royal White x White Dorper	2	F	POS	POS	0
TR-4106	TR	White Dorper x Dorcet	5	F	POS	POS	3 (SI)

Table S1a Cases: List of cases (N=9) with positive fecal qPCR results and evidence of tissue infection.

Sample ID	Origin Flock	Breed	Age at Necropsy (years)	Gender (M/F)	Fecal (q)PCR	Tissue (q)PCR	Acid-Fast Histopathology
C257 (C1)	M	Kattapakkam Red	6	F	POS	NEG	0
C168	M	Kattapakkam Red	7	F	POS	NEG	0
C374	M	Kattapakkam Red	5	F	POS	NEG	0
C495	M	Kattapakkam Red	2	F	POS	NEG	0
C424	M	Kattapakkam Red	5	F	POS	NEG	0
C699	M	Kattapakkam Red	2	M	POS	NEG	0
JD254	M	Kattapakkam Red	6	F	POS	NEG	0

JD522	M	Kattapakkam Red	4	F	POS	NEG	0
JD660	M	Kattapakkam Red	2	F	POS	NEG	0
JD803	M	Kattapakkam Red	2	M	POS	NEG	0
MJD347	M	Kattapakkam Red	6	F	POS	NEG	0
MJD533	M	Kattapakkam Red	4	F	POS	NEG	0
MJD637	M	Kattapakkam Red	3	F	POS	NEG	0
MJD663	M	Kattapakkam Red	2	M	POS	NEG	0
MJD826	M	Kattapakkam Red	2	M	POS	NEG	0
MJD890	M	Kattapakkam Red	2	M	POS	NEG	0
MJD 790	M	Kattapakkam Red	2	M	POS	NEG	0
MJD 741	M	Kattapakkam Red	3	F	POS	NEG	0
MJD 653	M	Kattapakkam Red	5	F	POS	NEG	0
M375	M	Kattapakkam Red	5	F	POS	NEG	0
M437	M	Kattapakkam Red	2	F	POS	NEG	0
M510	M	Kattapakkam Red	3	F	POS	NEG	0
M724	M	Kattapakkam Red	2	F	POS	NEG	0
M412	M	Kattapakkam Red	5	F	POS	NEG	0
M616	M	Kattapakkam Red	3	F	POS	NEG	0

Table S1b Controls: List of controls (N=25) fecal qPCR positive but without evidence of tissue infection.
