# Supplementary material for: Retrospective Single Nucleotide Polymorphism Analysis of Host Resistance and Susceptibility to Ovine Johne’s Disease Using Restored FFPE DNA
Source: Int J Mol Sci. 2024 Jul 15;25(14):7748. doi: 10.3390/ijms25147748 (PMC11276633; doi:10.3390/ijms25147748)
Supplement: Supplementary file 1 [file ijms-25-07748-s001.zip › S2 IJMS.pdf]

Supplementary Table S2: FFPE Gene Functions

S2 Table FFPE Gene Functions: Gene functions associated with identified significant SNPs from GWAS. All genes and functions were adapted from NCBI Genome.

**Chromosome OAR**	**NCBI SNP ID**	**Gene Symbol**	**Gene Name and Function**	**SNP location in gene**
1	rs401362015	RAB5A	RAB5A member RAS oncogene family<br><br>Enables GDP, GTP, and GTPase activity. (wnt signaling, autophagosome)	Within gene
1	rs407060336	TMCO1	Transmembrane and coiled-coil domains 1<br><br>Encoded transmembrane protein, involved in calcium channel activity.	Within gene
1	rs410166885	N/A	N/A	N/A
3	rs428083866	ANTXR1	ANTXR cell adhesion molecule 1<br><br>Encodes type I transmembrane protein, docking receptor for *Bacillus anthracis* toxin.	Within gene
3	rs401844951	ANKS1B	Ankyrin repeat and sterile alpha domain containing 1B<br><br>Envolved in brain development and pathogenesis of Alzheimer's disease.	Within gene
3	rs399773060	CFAP54	Cilia and flagella associated protein 54<br><br>Predicted involvement in cilium movement involved in cell motility.	Within gene
4	rs406625389	LOC101104484<br><br><br><br>PUS7	Nucleotide triphosphate diphosphatase NUT15-like<br><br>Pseudouridine synthase 7<br><br>Enables enzyme binding activity and pseudouridine synthesis.	10,635bp upstream<br><br><br><br>63,974bp upstream

		RINT1<br><br>ATXN7L1<br><br>SRPK2<br><br>KMT2E	RAD50 interactor 1<br><br>Encoded protein involved in regulation of cell cycle progression, role in trafficking cellular cargo from endosome.<br><br>Ataxin 7 like 1<br><br>SRSF protein kinase 2<br><br>Involved in nucleic acid metabolism, regulation of viral replication, and involved in innate immune response.<br><br>Lysine methyltransferase 2E<br><br>Member of the myeloid/lymphoid or mixed-lineage leukemia (MLL) family of proteins, overexpression inhibits cell cycle progression.	108,255bp upstream<br><br>282,590bp upstream<br><br>140,876bp downstream<br><br>306,504bp downstream
24	rs55627888	TMEM270<br><br>METTL27<br><br>CLDN4	Transmembrane protein 270<br><br>Methyltransferase like 27<br><br>Claudin 4<br><br>Encodes a member of the Claudin family of integral membrane proteins within epithelial cell tight junctions. High affinity receptor for *Clostridium perfringens* enterotoxin<br><br>Claudin 3<br><br>Member of the Claudin family of integral membrane proteins, a component of tight junction strands.	23,765bp upstream<br><br>53,426bp upstream<br><br>57,199bp upstream

		CLDN3<br><br>ABHD11<br><br>STX1A<br><br>BUD23<br><br>VPS37D	Involved in host response to gram positive bacteria.<br><br>Abhydrolase domain containing 11<br><br>Syntaxin 1A<br><br>Encodes member of syntaxin superfamily of nervous system-system specific proteins. Key molecule in ion channel regulation and synaptic exocytosis.<br><br>BUD23 rRNA methyltransferase and ribosome maturation factor<br><br>Encoded protein suggested to be involved in DNA methylation.<br><br>VPS37D subunit ESCRT-I<br><br>Predicted to be involved in protein targeting to vacuole, and ubiquitin–dependent protein catabolic processes.<br><br>MLX interacting protein like<br><br>Codes for a transcription factor, promotes triglyceride synthesis genes.<br><br>Transducin beta like 2<br><br>Encodes member of the beta-transduction protein family, involved in regulation of intracellular signaling.<br><br>BAF chromatin remodeling complex subunit BCL7B<br><br>Gene encodes member of the BCL7 family of proteins, involved in **wnt signaling pathway?**	87,600 bp upstream<br><br>112,155bp upstream<br><br>136,293bp upstream<br><br>147,986bp upstream<br><br>164,464bp upstream

		MLXIPL	Bromodomain adjacent to zinc finger domain 1B<br><br>Encodes member of bromodomain protein family, involved in chromatin-dependent regulation of transcription.	203,427bp upstream
		TBL2	Frizzled class receptor 9<br><br>Encodes for a 7-transmembrane protein that function as receptors for Wnt signaling proteins.	225,892 bp upstream
		BCL7B	FKBP prolyl isomerase family member 6<br><br>Encodes a cis-trans peptidyl-prolyl isomerase proposed to be involved in immunoregulation and basic cellular processes.<br><br>E3 ubiquitin-protein ligase TRIM50<br><br>NOP2/Sun RNA methyltransferase 5<br><br>Member of a conserved protein family thought to function as methyltransferases.	241,257bp upstream
		BAZ1B	POM121 transmembrane nucleoporin C<br><br>Predicted to encode protein involved in nuclear localization sequence binding activity.	279,959bp upstream
		FZD9	E3 ubiquitin-protein ligase TRIM50<br><br>NOP2/Sun RNA methyltransferase 5	310,853bp upstream

		FKBP6<br><br>TRIM50<br><br>NSUN5<br><br>POM121C	Member of a conserved protein family thought to function as methyltransferases.<br><br>POM121 transmembrane nucleoporin C<br><br>Predicted to encode protein involved in nuclear localization sequence binding activity.	372,676bp upstream<br><br>392,672bp upstream<br><br>401,307bp upstream<br><br>418,031bp upstream
26		KCNU1	Potassium calcium-activated channel subfamily U member 1<br><br>Encodes a voltage-gated ion channel, involved in outward potassium ion flow during plasma membrane hyperpolarization in sperm.	Within gene
26		THAP1	THAP domain containing 1<br><br>Encoded protein contains a conserved THAP DNA-binding	18,680bp upstream

			domain, acts as a proapoptotic factor.	
		RNF170	Ring finger protein 170<br><br>Encoded protein functions as an E3 ubiquitin ligase.	48,451bp upstream
		HOOK3	Hook microtubule tethering protein 3<br><br>Encodes a cytosolic coiled-coil protein involved in microtubule mediated binding to organelles.	109,402bp upstream
		FNTA	Farnesyltransferase, CAAX box, alpha<br><br>Gene encodes alpha subunit of a prenyltransferase group of transferases.	189,976bp upstream
		POMK	Protein O-mannose kinase<br><br>Encodes protein involved in formation of transmembrane linkages between extracellular matrix and exoskeleton, O-linked carbohydrate unit used by some pathogens for host entry.	214,256bp upstream
		HGSNAT	Heparan-alpha-glucosaminidine N-acetyltransferase<br><br>Encodes a lysosomal acetyltransferase involved in lysosomal degradation of heparin sulfate.	248,466bp upstream
			Integrator complex subunit 10	

		INTS10<br><br>CHRNA6<br><br>CHRNB3<br><br>SMIM19<br><br>SLC20A2	Encodes subunit of the Integrator complex involved in 3-prime end processing of small nuclear RNAs.<br><br>Cholinergic receptor nicotinic alpha 6 subunit<br><br>Encodes an alpha subunit of neuronal nicotinic acetylcholine receptors that function as ion channels.<br><br>Cholinergic receptor nicotinic beta 3 subunit<br><br>Encodes member of the nicotinic acetylcholine receptors (nAChRs) that function as ligand-gated ion channels that mediate fast signal transmission at synapses.<br><br>Small integral membrane protein 19<br><br>Solute carrier family 20 member 2<br><br>Encodes a type 3 sodium-dependent phosphate symporter, involved in phosphate homeostasis and susceptibility to viral infection as a gamma-retroviral receptor.<br><br>Voltage dependent anion channel 3<br><br>Encodes a voltage-dependent anion channel (VDAC), involved in mitochondrial permeability in apoptosis.<br><br>DNA polymerase beta<br><br>Encodes a DNA polymerase involved in base excision and repair.<br><br>Inhibitor of nuclear factor kappa B kinase subunit beta	285,309bp upstream<br><br>22,747bp downstream<br><br>55,225bp downstream<br><br>171,381bp downstream<br><br>233,424bp downstream

		VDAC3<br><br>POLB<br><br>IKBKB<br><br>PLAT	Encoded protein phosphorylates the inhibitor in the inhibitor/NF-kappa-B complex resulting in activation of NF-kappa-B.<br><br>Plasminogen activator tissue type<br><br>Encodes a secreted serine protease responsible for conversion of plasminogen to plasmin.	299,120bp downstream<br><br>340,954bp downstream<br><br>398,692bp downstream<br><br>486,876bp downstream
