# Supplementary material for: Retrospective Single Nucleotide Polymorphism Analysis of Host Resistance and Susceptibility to Ovine Johne’s Disease Using Restored FFPE DNA
Source: Int J Mol Sci. 2024 Jul 15;25(14):7748. doi: 10.3390/ijms25147748 (PMC11276633; doi:10.3390/ijms25147748)
Supplement: Supplementary file 1 [file ijms-25-07748-s001.zip › S3 IJMS.pdf]

Supplementary Table S3: Full List of KEGG Pathways and Genes

Table S3: (N = 74) total Ovine KEGG Pathways Identified using 16 physiologically relevant genes based upon known MAP-host pathophysiology.

Pathway Number	KEGG Pathway ID	Pathway Name	Genes
1	**oas04014**	Ras signaling pathway	RAB5A
			IKBKB
2	**oas04144**	Endocytosis	RAB5A
			VPS37D
3	oas04145	Phagosome	RAB5A
4	oas04962	Vasopressin-regulated water reabsorption	RAB5A
5	oas05014	Amyotrophic lateral sclerosis	RAB5A
6	**oas05022**	Pathways of neurodegeneration - multiple diseases	RAB5A
			STX1A
			FZD9
			VDAC3
7	**oas05132**	Salmonella infection	RAB5A
			IKBKB
8	oas05146	Amoebiasis	RAB5A
9	oas05152	Tuberculosis	RAB5A
10	**oas04621**	NOD-like receptor signaling pathway	ANTXR1
			IKBKB

			VDAC3
11	**oas04022**	cGMP-PKG signaling pathway	KCNU1
			VDAC3
12	oas04270	Vascular smooth muscle contraction	KCNU1
13	**oas04911**	Insulin secretion	KCNU1
			STX1A
14	oas01523	Antifolate resistance	IKBKB
15	oas04010	MAPK signaling pathway	IKBKB
16	oas04062	Chemokine signaling pathway	IKBKB
17	oas04064	NF-kappa B signaling pathway	IKBKB
18	oas04068	FoxO signaling pathway	IKBKB
19	**oas04150**	mTOR signaling pathway	IKBKB
			FZD9
20	oas04151	PI3K-Akt signaling pathway	IKBKB
21	oas04210	Apoptosis	IKBKB
22	oas04380	Osteoclast differentiation	IKBKB
23	oas04620	Toll-like receptor signaling pathway	IKBKB
24	oas04622	RIG-I-like receptor signaling pathway	IKBKB

25	oas04623	Cytosolic DNA-sensing pathway	IKBKB
26	oas04625	C-type lectin receptor signaling pathway	IKBKB
27	oas04657	IL-17 signaling pathway	IKBKB
28	oas04658	Th1 and Th2 cell differentiation	IKBKB
29	oas04659	Th17 cell differentiation	IKBKB
30	oas04660	T cell receptor signaling pathway	IKBKB
31	oas04662	B cell receptor signaling pathway	IKBKB
32	oas04668	TNF signaling pathway	IKBKB
33	oas04722	Neurotrophin signaling pathway	IKBKB
34	oas04910	Insulin signaling pathway	IKBKB
35	oas04920	Adipocytokine signaling pathway	IKBKB
36	oas04931	Insulin resistance	IKBKB
37	**oas05010**	Alzheimer disease	IKBKB
			VDAC3
			FZD9
38	oas05135	Yersinia infection	IKBKB

39	oas05145	Toxoplasmosis	IKBKB
40	**oas05160**	Hepatitis C	IKBKB
			CLDN4
			CLDN3
41	**oas05161**	Hepatitis B	IKBKB
			VDAC3
42	oas05164	Influenza A	IKBKB
43	oas05167	Kaposi sarcoma-associated herpesvirus infection	IKBKB
44	oas05171	Coronavirus disease	IKBKB
45	**oas05200**	Pathways in cancer	IKBKB
			FZD9
46	oas05220	Chronic myeloid leukemia	IKBKB
47	oas05235	PD-L1 expression and PD-1 checkpoint pathway in cancer	IKBKB
48	oas05417	Lipid and atherosclerosis	IKBKB
49	oas04130	SNARE interactions in vesicular transport	STX1A
50	oas04721	Synaptic vesicle cycle	STX1A
51	**oas05016**	Huntington disease	STX1A
			VDAC3
52	**oas04080**	Neuroactive ligand-receptor interaction	CHRNA6
			CHRNB3

53	oas04725	Cholinergic synapse	CHRNA6
54	oas04310	Wnt signaling pathway	FZD9
55	oas04390	Hippo signaling pathway	FZD9
56	oas04550	Signaling pathways regulating pluripotency of stem cells	FZD9
57	oas04934	Cushing syndrome	FZD9
58	oas05205	Proteoglycans in cancer	FZD9
59	oas05225	Hepatocellular carcinoma	FZD9
60	oas05226	Gastric cancer	FZD9
61	oas04371	Apelin signaling pathway	PLAT
62	oas04610	Complement and coagulation cascades	PLAT
63	oas05202	Transcriptional misregulation in cancer	PLAT
64	**oas04514**	Cell adhesion molecules	CLDN4
			CLDN3
65	**oas04530**	Tight junction	CLDN4
			CLDN3
66	**oas04670**	Leukocyte transendothelial migration	CLDN4
			CLDN3

67	oas04020	Calcium signaling pathway	VDAC3
68	oas04216	Ferroptosis	VDAC3
69	oas04217	Necroptosis	VDAC3
70	oas04218	Cellular senescence	VDAC3
71	oas04613	Neutrophil extracellular trap formation	VDAC3
72	oas04979	Cholesterol metabolism	VDAC3
73	oas05020	Prion disease	VDAC3
74	oas05203	Viral carcinogenesis	VDAC3
